# Low-Power Light Guiding and Localization in Optoplasmonic Chains Obtained by Directed Self-Assembly

**DOI:** 10.1038/srep22621

**Published:** 2016-03-02

**Authors:** Wonmi Ahn, Xin Zhao, Yan Hong, Björn M. Reinhard

**Affiliations:** 1 Department of Chemistry and The Photonics Center, Boston University, Boston, MA 02215, United States

## Abstract

Optoplasmonic structures contain plasmonic components embedded in a defined photonic environment to create synergistic interactions between photonic and plasmonic components. Here, we show that chains of optical microspheres containing gold nanoparticles in their evanescent field combine the light guiding properties of a microsphere chain with the light localizing properties of a plasmonic nanoantenna. We implement these materials through template guided self-assembly and investigate their fundamental electromagnetic working principles through combination of electromagnetic simulations and experimental characterization. We demonstrate that optoplasmonic chains implemented by directed self-assembly achieve a significant reduction in guiding losses when compared with conventional plasmonic waveguides and, at the same time, retain the light localizing properties of plasmonic antennas at pre-defined locations. The results reinforce the potential of optoplasmonic structures for realizing low-loss optical interconnects with high bandwidth.

Optical energy capture, transfer, and conversion are ubiquitous and efficient in nature and form the basis for life on Earth. Energy transport is also crucial in man-made electronic devices, but after decades of miniaturization thermal losses and signal delays at conventional copper-based interconnects create now fundamental physical limitations that challenge both a further performance enhancement and adequate energy efficiency for the next generation of electronics^1^. The ability to manipulate light on deeply subdiffraction limit length scales with the help of plasmonic nanocircuitries has the potential to overcome some of these limitations and to bridge the fields of electronics and photonics^2^,^3^,^4^,^5^. Low light guiding efficiencies in metallic nanostructures complicate, however, the application of plasmonic circuitries, for instance, for networking distributed electronic architectures.

Plasmonic waveguides^6^,^7^,^8^ support propagating surface plasmons that decay exponentially^9^ with typical attenuation levels of 1.4 dB (at 830 nm) and 1.8 dB (at 760 nm) per micron in chemically synthesized silver^10^ and gold^11^ nanowires, respectively, and 1.7 dB per micron (at 800 nm) in a thermally evaporated gold strip^12^. Some applications require both light guiding and subsequent localization in plasmonic gap structures^13^,^14^ to enhance light-matter interactions^15^,^16^,^17^,^18^. Possible device designs based on near-field coupled plasmonic antennas suffer from the fact that gaps between plasmonic nanostructures^19^,^20^ further increase propagation losses. In a linear chain of 25 nm radius silver particles separated by an edge-to-edge distance of 25 nm an attenuation of ~6 dB^21^ and 4.8 dB^22^ per micron was measured. Since the high losses in plasmonic architectures challenge the utility of these devices, strategies for off-setting or minimizing the losses are currently being explored. These strategies include active loss compensation based on optical gain media^23^,^24^, as well as novel plasmonic light guiding concepts^9^,^25^, alternative plasmonic materials^26^,^27^, and enhanced fabrication strategies that reduce surface roughness^28^,^29^. Despite significant advancements, the realization of both efficient long-distance guiding and nanofocusing in plasmonic structures is still met by fundamental challenges.

These challenges motivate the development of metallo-dielectric hybrid architectures in which plasmonic nanostructures with small mode volumes but modest Q-factors are combined with low-loss photonic structures that can efficiently guide, switch, and route the light over longer length scales to finally refocus it into nanoscale hot-spots created by plasmonic antennas for detection in the near- or far-field^30^. Diverse photonic-plasmonic hybrid approaches are conceivable for this purpose, including the integration of plasmonic antennas into photonic crystals^31^, silicon waveguides^32^, or dielectric micro ring resonators^33^ and toroidal microcavities^34^. However, for most of these hybrid devices reliable fabrication approaches are missing, requiring instead the manipulation of the metal and dielectric components using a micromanipulator or scanning probe^35^,^36^. Furthermore, it is also often difficult to miniaturize these hybrid structures and embed them into an on-chip platform as they require external light couplers, detectors, or other bulky components^37^,^38^,^39^,^40^.

We implement, test, and characterize here an alternative approach that is compatible with on-chip integration, does not require bulky peripherals, and that benefits from the efficient light circulation in optical microcavities (OMs) to enhance light-matter interactions. Our approach combines networks of dielectric microspheres with metallic nanostructures located at discrete locations in the evanescent field of the OMs. These so-called optoplasmonic chains aim to combine the efficient light guiding properties of OM chains with the light localizing properties of plasmonic antennas at defined locations. Importantly, these structures can be fabricated in a rational fashion by established template guided self-assembly strategies^41^,^42^ that use metal tipped posts to position plasmonic antennas in the evanescent field of the OMs. In this manuscript we combine electromagnetic simulations and fluorescence spectroscopy to characterize light guiding, localization, and energy transfer in optoplasmonic chains.

## Results and Discussion

### Design of Optoplasmonic Chains

Chains of dielectric microspheres have been investigated in detail due to their utility as efficient optical waveguides^43^,^44^. The individual microspheres in the chains are usually touching (in the case of glass microspheres^45^) or connected by micro-joints (in the case of polystyrene (PS) microspheres^46^), unless the dimensions of the sphere-to-sphere gaps were carefully controlled by substrate micropatterning^47^,^48^ or bead assembling through micromanipulation^49^. The micro-joints in the polymeric beads result from polymer swelling and subsequent material reflow, which are irreversible processes that perturb optical responses of the chain^50^. In general, several complementing mechanisms exist for energy transfer in the conventional coupled OM chains, including evanescent whispering gallery mode (WGM) coupling^51^, photonic nanojet induced mode (NIM) coupling between adjacent OMs^43^,^52^, and far-field type light propagation^50^. Waveguides based on weak coupling between otherwise individual cavity modes of dielectric microspheres^53^ or microring resonators^54^ are referred to as coupled-resonator optical waveguides (CROWs).

**Figure 1:**
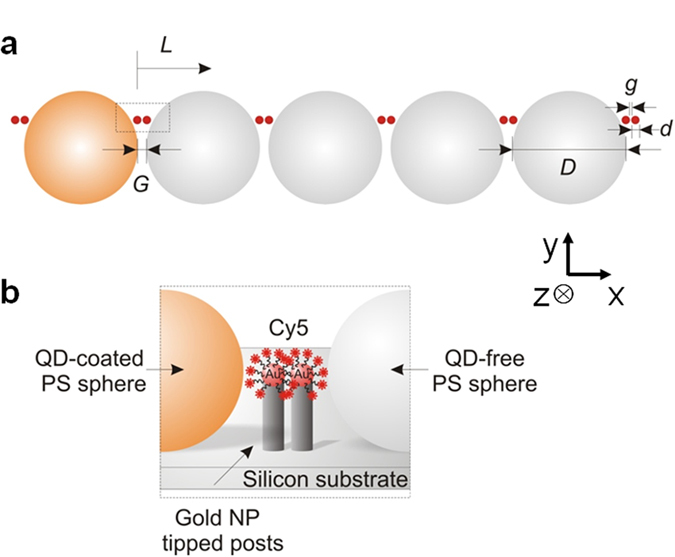
A schematic diagram of the optoplasmonic chain. (**a**) A top-down view of the chain containing 5 WGM sustaining OMs, each surrounded by 4 gold nanoparticles located at the evanescent field of the microspheres. The first OM (orange) is coated with QDs (emission wavelength at 605 nm), acting as an on-chip light source in the chain of QD-free OMs (gray). *D* and *d* refer to a diameter of the microsphere and the gold nanoparticle, respectively. *G* and *g* refer to a gap between OMs and a gap between gold nanoparticles, respectively. A distance from the light source, *L*, is measured from the QD-coated OM. (**b**) A side view of the boxed area in (**a**) where gold nanoparticle tipped posts are located in the evanescent field of the OMs but off-axis of the OM chain. Gold nanoparticles are coated with Cy5-dyes using 30 bp DNA strands.

A schematic diagram of the proposed optoplasmonic chain is shown in Fig. 1. A quantum dot (QD) functionalized PS microsphere (orange) acted as an integrated light source. The general idea of the optoplasmonic chain is that the QD-coated bead induces WGMs in the adjacent microspheres that then propagate across the chains of coupled OMs through a CROW-like coupling mechanism. A fraction of the guided energy gets localized at the different plasmonic antennas where the locally enhanced *E*-field allows for an efficient excitation of the Cy5 acceptor dyes microns away from the light source. The exact distance *L* was measured from the QD-coated OM (Fig. 1a).

We investigated chains containing up to 5 WGM sustaining OMs (diameter, *D*), each surrounded by 4 metallic nanoparticles (NPs; diameter, *d*, and dimer gap, *g*) at pre-defined locations (Fig. 1a). The microsphere chain defines the axis of the OM waveguide, and the position of the pillars was chosen to achieve a close spacing between the microspheres (typically 30–50 nm) and to position Cy5-coated plasmonic antennas in the evanescent field of the OMs but off-axis of the OM chain. The off-axis position was chosen to minimize the perturbation of CROW-mediated coupling between OMs and, at the same time, achieve a short spacing between the microspheres for an efficient light guiding along the chain axis. A silicon wafer was used as a supporting substrate both in the experiment and simulation.

### Electromagnetic Simulation of the Near Field in Optoplasmonic Chains

**Figure 2:**
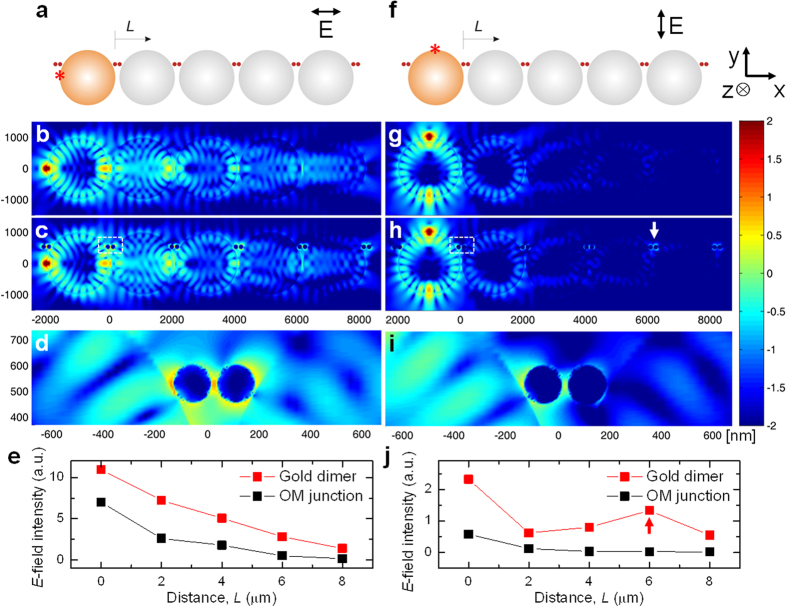
*E*-field intensity distributions in the optoplasmonic chain at the wavelength, λ = 650 nm. A dipole emitter is located on (**a–e**) and off (**f–j**) the axis of the OM chain. (**a,f**) Schematic diagrams of the optoplasmonic chains that consist of the coupled OMs (*D* = 2 μm, *G* = 50 nm) and gold dimers (*d* = 150 nm, *g* = 20 nm). The dipole emitter locations are indicated with red stars. *E*-field intensity maps in log(|E|^2^/|E_0_|^2^) are shown for metal-free OM chains (**b,g**) and metal containing optoplasmonic chains (**c,h**). (**d,i**) show the magnified OM–gold dimer interfaces at *L* = 0 for corresponding dipole locations. (**e,j**) are *E*-field intensity values measured from (**c,h**), respectively, as a function of *L*. They are peak values obtained from 400 × 400 nm^2^ areas centered at gold dimers (red) and OM junctions (black). *E*-field intensity distributions simulated via Lumerical software (Ver. 8.7.0.; www.lumerical.com) were generated into images using Matlab (Ver. 7.11.0.584 R2010b; http://www.mathworks.com/products/matlab/).

In the first step of our systematic analysis of the optoplasmonic chain, we analyze the *E*-field intensity along the optoplasmonic chains at a fixed wavelength of 650 nm using finite difference time domain (FDTD) simulations. The bandwidth of the optoplasmonic chains presented in this work lies between 600 and 850 nm, providing the most efficient photonic-plasmonic mode coupling at 650 nm. For a waveguide structure where the light source is a quantum emitter-functionalized OM, the efficiency of the respective light guiding mechanism also depends on the position and orientation of the emitter around the OM. While in our experimental implementation of the optoplasmonic chain we average over many different emitter arrangements, the electromagnetic simulations make it possible to systematically investigate the impact of the emitter location and orientation. The contribution from WGMs to the energy transport across the chain also depends on the orientation and location of the exciting dipole emitter. Representative examples for *E*-field intensity patterns associated with different excitation configurations are shown in a log scale in Fig. 2 for dipoles oriented perpendicular to the OM surface, which couple primarily to WGM guided modes. If a dipole emitter is located on the axis of the OM chain (Fig. 2a–e) it couples to TM modes on orbits in the xy- and xz-plane which propagate along the OM chain (Fig. 2b)^48^. The OM chains act as coupled spherical lenses, forming increased intensity spots with 2D periodicity^55^. The *E*-field orientation perpendicular to the OM surface in case of the TM modes ensures an *E*-field component along the long axis of the NP dimer antennas placed ~350 nanometers away from the OM chain axis (Fig. 2c). Consequently, this dipole orientation achieves a significant *E*-field localization on the metal NPs along the entire chain length (Fig. 2c). For dipoles oriented perpendicular to the OM surface but off-axis (Fig. 2f–j) the coupling efficiency to guided modes along the OM chain is lower, as only the TM modes with an orbit in the xy-plane can propagate along the chain. The *E*-field intensity maps for tangential orientations of dipoles located on- and off-axis are summarized in Figure S1.

The effect of the plasmonic nanoparticles on the near-field in optoplasmonic chains is illustrated by a comparison of the *E*-field intensity maps of (**b**) the metal-free OM chain and (**c**) the metal-containing optoplasmonic chain with an emitter located on the microsphere chain axis. The optoplasmonic chain in (**c**) clearly shows *E*-field localization around gold dimers (*d* = 150 nm; *g* = 20 nm) for all antennas of the chain. Interestingly, especially for the last two OMs of the optoplasmonic chain the *E*-field intensity in and around the OMs is decreased when compared with the metal-free OM chain. This observation indicates a redistribution of optical power from the OMs to the plasmonic components. The excitation of the NP dimer antennas along the OM chain is mainly through the evanescent tails of WGMs in the individual OMs. Figure 2d contains a magnified *E*-field intensity map of the white boxed area in (**c**), which illustrates how the gold dimer refocuses the field around the OMs. The exact *E*-field intensity distribution around the plasmonic antenna at a given wavelength depends on the location and orientation of the nanoparticle dimer relative to the OM (as shown in Fig. 2), and can also be modulated by variation of the wavelength (as will be shown below). For the optoplasmonic chain containing an off-axis located emitter as an excitation source the *E*-field intensities are–as expected–overall lower, but the gold nanoparticles still capture the evanescent *E*-field from the OMs and refocus it as shown in Fig. 2h,i.

Importantly, for both simulated excitation geometries the *E*-field intensity localized in the vicinity of the gold dimers is higher than the *E*-field intensity in the OM junctions due to photonic-plasmonic mode coupling. This is shown in Fig. 2e,j where we plot the peak *E*-field intensity values obtained from 400 × 400 nm square areas centered at each gold dimer and OM junction. Overall, the *E*-field intensity decreases at both monitored positions as a function of a distance from the light source, *L*. However, the peak *E*-field intensity is higher around the plasmonic antenna than in the OM junctions for all *L* due to the strong *E*-field localization provided by the plasmonic antennas. The unusually high *E*-field intensity of the gold dimer at 6 μm in Fig. 2h,j (marked with arrows) indicates that the local intensity of the hot-spot is further modulated by other effects, in particular the relative orientation of the dimer and the WGM field distribution close to the OM surface (*vide infra*).

**Figure 3:**
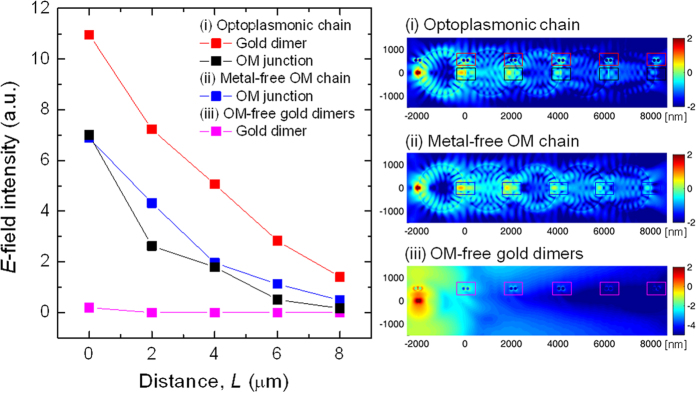
Electric field intensities in the simulated (**i**) optoplasmonic chain (*G* = 50 nm), (**ii**) metal-free OM chain (*G* = 0 nm), and (**iii**) OM-free gold dimers, as a function of *L*, a distance from the light source. They are peak values isolated from 400 × 400 nm^2^ areas centered at each gold dimer and OM junction. Inset shows *E*-field maps of each structure. Lumerical (Ver. 8.7.0.; www.lumerical.com) and Matlab (Ver. 7.11.0.584 R2010b; http://www.mathworks.com/products/matlab/).

One unique aspect of the optoplasmonic chain is that it combines the guiding of electromagnetic energy over several microns with a subsequent localization into a nanoscale volume where the locally enhanced electromagnetic field can interact with quantum emitters. The mechanism and efficiency of energy transfer from one OM to the next therefore strongly depends on the gap width, *G*, between the OMs. We performed FDTD simulations to evaluate the influence of a 50 nm gap width on the light guiding efficiency of the optoplasmonic chains (Fig. 3) and included gapless OM chains as a benchmark (Inset of Fig. 3, metal-free OM chain). A further reference structure necessary to evaluate the gain in functionality in optoplasmonic chains is that of gold dimers spaced at an equal distance as the ones in the optoplasmonic chain but missing the OMs (Inset of Fig. 3, OM-free gold dimers). The dipole emitter is located on the chain axis in all three cases. As shown in the *E*-field intensity plots measured in different structures (Fig. 3), the guiding efficiency of the OMs in the optoplasmonic chain (black) is comparable to that of the touching OMs (blue), confirming that the 50 nm gap is small enough to allow for an efficient transfer of optical energy along the optoplasmonic chain. Importantly, the *E*-field intensity enhancement provided by the gold nanoparticle dimers in the optoplasmonic chain (red) surpasses that of the metal-free OM chain (blue) and OM-free gold dimers (magenta). The CROW coupling mechanism in the optoplasmonic chain generates *E*-field intensity around the entire circumference of the coupled OMs, resulting in an excitation of plasmons in the off-axis positioned metal nanoparticles. We conclude that by placing metal nanoparticles at the spatially well-controlled evanescent fields of the WGM resonators, the optoplasmonic chain efficiently combines CROW-mediated energy guiding and noble metal nanoparticle mediated light localization to achieve a strong localization of electromagnetic energy into a truly nanoscale volume located microns away from the emitter.

### Powerflow around Gold Dimers in Optoplasmonic Chains

**Figure 4:**
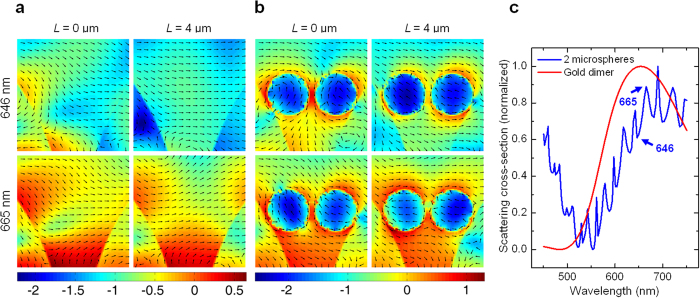
Poynting vector intensity distributions of the metal-free OM chain (**a**) and gold dimer including optoplasmonic chain (**b**) at given locations of *L* = 0 and 4 μm. The spectral locations of 646 nm (WGM dip) and 665 nm (WGM peak) are selected based on the simulated scattering spectra shown in (**c**) for 2 OMs (blue) and gold dimer (red). *d* = 150 nm, *g* = 20 nm, OM–gold nanoparticle gap = 5 nm. Lumerical (Ver. 8.7.0.; www.lumerical.com) and Matlab (Ver. 7.11.0.584 R2010b; http://www.mathworks.com/products/matlab/).

A closer look at the *E*-field intensity maps in Fig. 2d,i reveals that the optoplasmonic chain is also capable of controlling the near-field around the metal nanoparticles and differs at different positions along the chain due to variations in the morphology of the electromagnetic field generated by the OM chain around the metal nanoparticle antenna. In addition, the field distribution in the optoplasmonic chain is strongly wavelength dependent. In order to describe the vector field of the optical powerflow around the gold dimers in detail, we performed time-averaged Poynting vector simulation using FDTD. Figure 4 shows the Poynting vector intensity distributions of a conventional metal-free chain of OMs (**a**) and a metal-containing optoplasmonic chain (**b**) at locations of *L* = 0 and 4 μm at selected wavelengths of 646 and 665 nm. We chose each wavelength as WGMs of two coupled OMs show a distinct peak at 665 nm and a dip at 646 nm as revealed in the simulated scattering cross-section spectra in Fig. 4c. The dipole emitter is located on the OM chain axis and is oriented perpendicular to the OM surface. In both investigated chain types the OMs are separated by 50 nm gaps.

In the OM chains the Poynting vector field and the *E*-field intensity distribution in the gaps are determined by the WGMs in the adjacent OMs (Fig. 4a). Importantly, the optical powerflow and *E*-field intensity maps are not identical but differ for different gap positions of *L* = 0 and 4 μm along the chain and at different wavelengths. A stronger flow and field intensity is obtained at the gap located closer to the dipole emitter and at the WGM peak frequency. Moreover, the positioning of the nanoparticle dimer close to the OM surface (Fig. 4b) has profound effects on the Poynting vector map. The metal nanoparticles redirect the local optical powerflow in their immediate vicinity, including the adjacent regions of the OM. Differences in the field and phase distribution between different gaps result in different spatial *E*-field distributions around the metal nanoparticles as shown in *L* = 0 and 4 μm of Fig. 4b. One aspect of special notice is that the excitation of the plasmonic structure through the evanescent field of the WGMs in the OM chains creates frequency-dependent asymmetric field patterns, which provides new opportunities for switching the spatial near-field distribution on the nanoscale by choice of the guided wavelength. At 665 nm, where WGMs have their maximum, the highest *E*-field intensity and the strongest powerflow around the gold dimer are achieved.

### Template-Guided Self-Assembly of Optoplasmonic Chains

**Figure 5:**
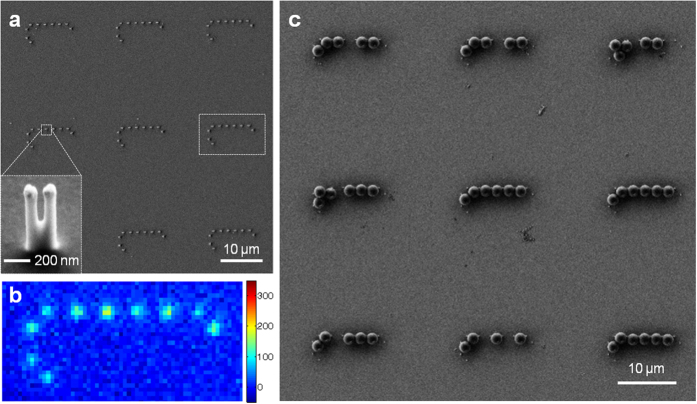
Optoplasmonic chains fabricated by template guided self-assembly. (**a**) A SEM image of gold nanoparticle tipped-posts in a 3 × 3 array. An inset SEM image shows individual gold nanoparticle tipped posts with gold nanoparticle dimers having a particle diameter (*d*) of 100 nm, inter-particle gap (*g*) of 40 nm, and post height (*h*) approximately 1 μm. (**b**) A fluorescence image of the Cy5-functionalized gold nanoparticle tipped posts; a boxed area in (**a**). (**c**) Optoplasmonic structures after insertion of OMs through convective self-assembly. We chose the completed optoplasmonic chains for the optical characterization. Scale bars are 10 μm in (**a,c**), and 200 nm in the inset of (**a**).

After characterizing the working principle of the optoplasmonic chains through electromagnetic simulations, we will in the next step implement these materials and test the predicted behavior experimentally. The experimental realization of the optoplasmonic chain requires the positioning of the plasmonic antennas at defined locations of the OM equatorial plane, which is 1 μm above the substrate surface. We applied a template guided self-assembly strategy to address this 3D fabrication challenge. First, we created gold nanoparticle tipped posts through the combination of electron beam lithography (EBL) and reactive-ion etching (RIE)^41^,^56^. Figure 5a shows a SEM image of typical gold nanoparticle tipped posts generated in a 3 × 3 array. The inset of Fig. 5a shows a 30°-tilted magnified SEM image of individual gold nanoparticle tipped posts with gold nanoparticles having a particle diameter (*d*) of 100 nm, inter-particle gap (*g*) of 40 nm, and post height (*h*) approximately 1 μm. A brush of 30 nucleotide long single-stranded DNA with a Cy5 dye on the 3′ end and gold binding thiol group on the 5′ end was assembled on the gold nanoparticles. The DNA served the purpose of a spacer to avoid the quenching of the organic dye in the immediate vicinity of the metal surface. The thickness of the 30 bp long DNA was measured as 13.5 nm on a 40 nm-thick gold film using an ellipsometer, which is large enough to avoid quenching^57^. Based on the footprint of the DNA of 15 nm^2^,^58^, the maximum number of dyes per particle is estimated as 2,000. The fluorescence image of the Cy5-functionalized gold nanoparticle tipped posts in Fig. 5b shows a clear signal at the position of the gold dimer posts, confirming that the dye functionalization was successful. The fluorescence intensities of the gold dimer tipped pillars are >8.5 times enhanced compared to that of thin gold films prepared under the same EBL and RIE conditions.

**Figure 6:**
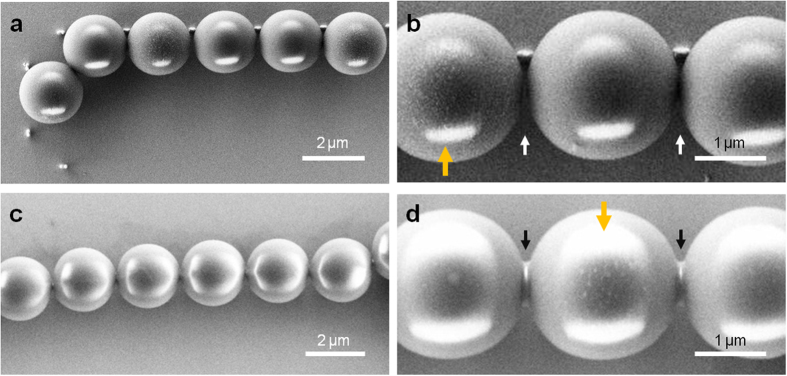
Comparison between the optoplasmonic chain (**a,b**) and the metal-free OM chain (**c,d**) in terms of micro-joint formation at OM junctions. Note that gold tipped silicon posts in the optoplasmonic chain separate OMs, creating 30–50 nm gap between them (**b**), white arrows). On the other hand, the post-free OM chain forms micro-joints between OMs through irreversible polymer swelling and material reflow (**d**), black arrows). The OMs indicated with orange arrows in (**b,d**) are the QD-coated OMs, clearly showing the surface coating with QDs. Scale bars are 2 μm in (**a,c**), and 1 μm in (**b,d**).

The binding sites defined by the posts were then filled with 2 μm diameter polystyrene beads through convective self-assembly (Fig. 5c). A careful control of the size of the binding sites and the height of pillars ensured the localization of the gold nanoparticle dimers in close vicinity to the microspheres. In most cases, the OM–gold edge-to-edge distance was measured on average ~20 nm, except for the last OMs in the chain, which usually creates a larger gap up to ~80 nm. At the same time, the posts were designed to separate microspheres with an average gap (*G*) of 30–50 nm to prevent the formation of irreversible polymer microjoints. A close-up SEM image of an individual optoplasmonic chain shows indeed that the microspheres are intact and separated at the junction areas (Fig. 6a,b; white arrows), while they appear fused in a control sample that lacks gold nanoparticle tipped posts (Fig. 6c,d; black arrows). One tenth of the PS microspheres (volume ratio) used to assemble the microsphere chains were coated with QDs with an emission maximum at 605 nm to serve as an on-chip light source in the optoplasmonic chains. The SEM images in Fig. 6b,d show that microspheres functionalized with QDs (orange arrows) have a rougher surface than other microspheres. When excited with a 420 nm LED, the QD-coated microsphere provided a bright orange fluorescence signal. For additional details about bead functionalization with QDs and convective self-assembly, please refer to the Methods section.

### Guiding Efficiency of Optoplasmonic Chains

**Figure 7:**
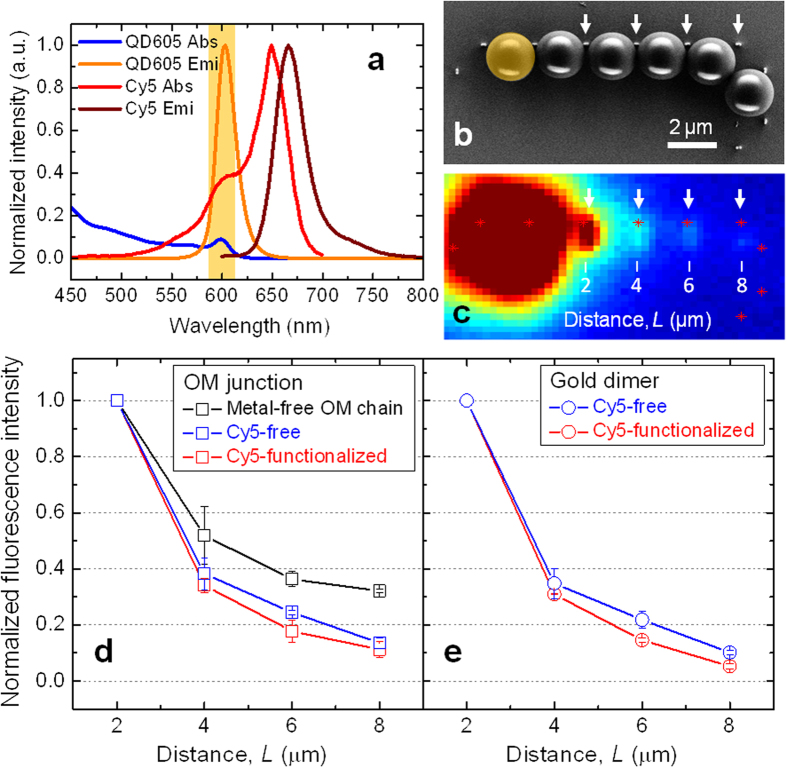
Guiding efficiency of the QD emitted light in the optoplasmonic chain. (**a**) The normalized absorption and emission spectra of QD 605 and Cy5. The light emitted from the QD 605 was detected with an emission filter (610/10 nm, shaded in orange). (**b**) A SEM (scale bar 2 μm) and (**c**) fluorescence image of an optoplasmonic chain in which the first microsphere is functionalized with QD 605 (colored in orange for better visualization), while the rest of PS microspheres are QD-free. Normalized fluorescence intensities measured (**d**) in the OM junctions and (**e**) at the gold dimers of the conventional CROW chain (black), Cy5-free optoplasmonic chain (blue), and Cy5-functionalized optoplasmonic chain (red).

The transfer of optical energy from QDs to remote Cy5 dyes *via* the optoplasmonic chains shown in Fig. 5 is a complex process that includes the capture and guiding of QD emitted light along the OM chain, light localization at the nanoparticle antennas, and the subsequent excitation and emission of Cy5. In the first step of an experimental characterization of the complex materials, we investigated the guiding efficiency of the QD emitted light under three different experimental conditions: i) conventional CROWs (no metal NPs but containing micro-joints, as shown in Fig. 6c,d), ii) Cy5-free optoplasmonic chain, and iii) Cy5-functionalized optoplasmonic chain. We chose QD-605 as QD in this study as its emission peak wavelength (λ_emi_, 605 nm) overlaps with a broad shoulder of Cy5 excitation wavelength (λ_exc_, 650 nm) with the minimal spectral cross-talk (Fig. 7a). The light emitted from the QD 605 was detected using a 610 nm emission filter (shaded in orange). Figure 7b contains the SEM image of the optoplasmonic chain whose fluorescence image is shown in Fig. 7c. A single QD-coated OM (shaded in orange) is located at the left end of the chain, while the same-sized QD-free spheres are aligned with an OM-to-OM gap separated by gold tipped nanopillars (white arrows). We evaluated the fluorescence intensity along the chain both in the OM gap (Fig. 7d) as well as at the positions of the plasmonic antennas (Fig. 7e), whose locations are exactly known from the SEM inspection of the sample after the fluorescence imaging. The locations of gold nanoparticle tipped pillars in the optoplasmonic chain are indicated with red stars in Fig. 7c. At these points the guided light is scattered either due to the refractive index difference associated with the microsphere gap or by excitation of plasmons in the metal nanoparticles. We characterized 5 different optoplasmonic chains with and without Cy5 through this correlated SEM/optical microscopy analysis, and the resulting fluorescence intensities are plotted as a function of distance (*L*) from the QD-coated OM.

Figure 7d shows that the chain of touching OMs (CROW structure, black) guides the QD-emitted light more efficiently than the optoplasmonic chains. It is known that micro-joints with a joint diameter corresponding to 1/2 of the wavelength act as an aperture, allowing NIM-guided modes to efficiently traverse through the micro-joints^50^. The diameter of micro-joints in our OM chain is measured as ~540 nm and, therefore, fulfills the requirement for efficient light propagation with relatively low losses. Assuming a simple exponential decay^50^ (*I* = *I*_*0*_*10*^*(−0.1)βL*^) where *L* is the distance, we derive an optical attenuation constant (*β*) of 0.51 ± 0.06 dB per micron from the CROW structure. The Cy5-free optoplasmonic chains show a higher attenuation of *β* = 0.86 ± 0.06 dB per micron. We ascribe the higher losses to the presence of 30–50 nm wide gaps between the individual microspheres *and* the addition of metal tipped pillars, which further reduces the light guiding efficiency due to photonic-plasmonic mode coupling in the chain. This is also indicated by the more rapid drop in fluorescence intensity as a function of separation in Fig. 7d (blue). We emphasize that while the excitation of plasmons is inevitably associated with dissipative losses, the optoplasmonic chain minimizes these losses by combining CROW waveguiding with plasmonic near-field localization at desired location. The success of this approach becomes clear by comparing optoplasmonic chains with one-dimensional arrays of near-field coupled metal nanoparticles^21^. The attenuation of *β* = 0.86 ± 0.06 dB per micron for the optoplasmonic chain is more than 7-fold lower than *β* = ~6 dB per micron^21^ for the plasmonic chain. Interestingly, the losses of the optoplasmonic chain increase further if Cy5 dye is conjugated to the plasmonic antennas, as indicated by *β* = 1.02 ± 0.13 per micron in Fig. 7d and 1.18 ± 0.02 dB per micron in Fig. 7e. Although the increase is small, it is significant and indicates energy transfer from the metal nanoparticle antennas to the attached dyes. Overall, these observations are consistent with the successful excitation of Cy5 dyes located micrometers away from the on-chip light source.

### Localization of Guided Light through Plasmonic Antennas and Excitation of Cy5

**Figure 8:**
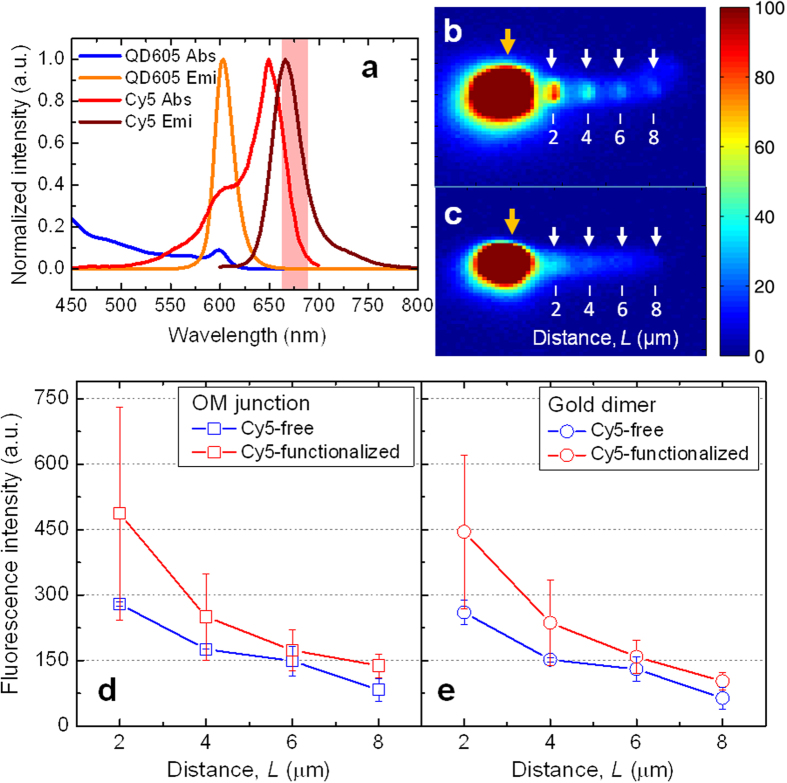
Direct emission of Cy5 dyes from the optoplasmonic chain. (**a**) Normalized absorption and emission spectra of QD 605 and Cy5 with an emission filter (680/22 nm, shaded in red). Fluorescence images of optoplasmonic chains: with (**b**) and without (**c**) Cy5 attachment on the gold dimers. Raw fluorescence intensities measured in the OM junctions (**d**) and at the gold dimers (**e**) of the optoplasmonic chains that are with (red) and without (blue) Cy5 attachment.

In the next step, we measured the direct Cy5 emission from the dye-functionalized optoplasmonic chains to further validate that the optoplasmonic chains not only guide the QD-emitted light through OMs but also focus it on the metal antennas to excite quantum emitters locally with high selectivity. These measurements were performed with a 420 nm LED for excitation and detecting fluorescence at 680 nm as indicated in Fig. 8a (shaded in red). Although the fluorescence intensity was overall very low, the images obtained from (**b**) Cy5 dye containing optoplasmonic chains and (**c**) control structures with identical geometry but free of dye showed some distinct differences. While the locations of the gold dimers and OM junctions are not detectable in the control (**c**), these points show detectable Cy5 signal intensity in (**b**) for *L* up to 8 μm (white arrows). The overall low intensity of the Cy5 emission in the Cy5-functionalized optoplasmonic chain can be explained in part by the low excitation power in our experiment and the directed emission of the Cy5 into the OM. The power transmitted in the optoplasmonic chain was estimated to be <10 pW (see Methods). Assuming significant coupling losses of light, realistic input power in the optoplasmonic chain is at least one order of magnitude lower. Also, consistent with previous studies by Bonod and co-workers^59^, our simulations (Figure S2) predict a preferential emission of the Cy5 dye into the near-by OM. The directed emission into the dielectric microsphere reduces the fraction of Cy5 emission detectable through far-field microscopy.

Figure 8d,e summarizes the measured fluorescence intensities at the locations of the OM junctions (**d**) and the gold dimers (**e**) for 5 optoplasmonic chains with (red) and without (blue) Cy5 dye tethered to the gold nanoparticles. The overall higher intensity for the Cy5 functionalized optoplasmonic chain is consistent with a successful excitation of the dye molecules through the QD emitted light guided by the OM chain and its relocalization by the plasmonic antennas where they excite the metal bound dyes.

In conclusion, the optoplasmonic chains investigated in this manuscript combine light guiding and subsequent localization at pre-defined locations and are compatible with low power applications. The investigated optoplasmonic chains used a single QD-coated microsphere as a light source, providing an input power of a few pW. The optoplasmonic chains generated by a template guided assembly approach demonstrate light guiding over several micrometers with an attenuation that is more than 7-fold lower than conventional chains of closely spaced metal nanoparticles although the optoplasmonic chain achieves a similar level of *E*-field localization at the attached plasmonic antennas. Efficient optoplasmonic networks that offer diverse functionalities for information transport and processing are of significant interest for a broad range of applications, including future low power electronics. We believe that the programmable structural complexity of optoplasmonic chains will be useful in diverse optoelectronic applications. Optoplasmonic chains offer a series of beneficial characteristics, such as high bandwidth, power efficiency and architectural flexibility, which are important requirements for improving the performance of future optical interconnects.

## Methods

### Fabrication of Optoplasmonic Chains

First, electron beam lithography (EBL) was used to define arrays of nano-dimers in a 200 nm thick poly(methylmethacrylate) (PMMA) layer spin-coated on a 1 × 1 cm^2^ silicon wafer chip. An input diameter (*d* = 150 nm) and inter-particle gap (*g* = 25 nm) of the dimer for the EBL were set to be larger and smaller, respectively, than the desired final sizes, because *d* value is decreased and *g* value is increased after reactive ion etching (RIE). After the EBL, an 80 nm thick gold film was evaporated on top of a 5 nm thick chromium adhesion layer, and finally 40 nm thick aluminum layer was deposited to prevent the gold layer from being etched by reactive gases. After the PMMA layer was lifted off, the substrate of the metal dimer arrays was brought into an inductively coupled-plasma RIE chamber (STS MPX/LPX system). A mixture of reactive gases of SF_6_ (60 standard cubic centimeters per minute, sccm) and C_4_F_8_ (160 sccm) carved down the substrate at a power of 1200 W for 5–8 min, resulting in the metal nanoparticle tipped silicon post arrays. The substrate was then immersed in an aluminum etchant solution for 1 min, rinsed with distilled water, and the fabrication of the gold nanoparticle tipped posts was complete.

The convective self-assembly method was used to integrate microspheres into the binding sites defined by gold nanoparticle tipped posts. For this, a mixture of QD-coated and -uncoated microspheres (1:10 volume ratio, total volume of 35 μL) was inserted into the gap that was created between the patterned substrate on the bottom and the same sized plain quartz substrate on the top separated by 350 μm thick small pieces of silicon chips (3 × 3 mm^2^) at the substrate corners. Upon evaporation of the microsphere suspension, the meniscus formed between two substrates slowly drags microspheres to the binding sites, resulting in the integration of the microspheres with the gold nanoparticles. This approach ensures that the gold nanoparticles are located in a close vicinity of microspheres without a direct metal attachment on the microsphere surface, which causes quenching of respective modes.

Control metal-free OM chains shown in Fig. 6c,d were prepared using a TEM grid (TedPella, Inc.) on unpatterned silicon substrates. First, a drop (~10 μL) of a mixed solution containing QD-coated and -uncoated microspheres was placed on top of a reference silicon wafer (1 × 1 cm^2^). The TEM grid made out of copper with an outer diameter of 3.05 mm was then placed flat on top of the drop until all liquid evaporates at room temperature. Since the TEM grid has square meshes (with a hole width of 7.5 μm and a bar width of 5 μm), liquid evaporates between these microsized holes of the TEM grid, resulting in the alignment of microspheres in a mesh structure. After evaporation of the liquid is complete the TEM grid was carefully removed using a pair of tweezers and the microsphere chains consisting of 5 OMs are used for the characterization. SEM images of the generated structures were taken using Zeiss SUPRA 40VP.

### Functionalization of the Polystyrene Microspheres with Quantum Dots

First, 200 μL of 2 μm diameter polystyrene microspheres (#4202 A, Thermo Fisher Scientific Inc.) was centrifuged down at 5.5 × 1000 rpm for 1 min to replace the stock liquid with biotinylated BSA (1 mg/mL; Thermo Fisher Scientific Inc.). After 90 min of incubation on a rotator the biotin-BSA functionalized polystyrene beads were washed with filtered PBS buffer (x0.1, pH 8) for 3 times to remove un-bound biotinylated BSA. The microspheres were then incubated with streptavidin conjugated quantum dots (Q10103MP, Thermo Fisher Scientific Inc.) at a concentration of 1 × 10^−2^ μM for 90 min on a rotator. We chose the quantum dot that has a narrow emission maximum at 605 nm and streptavidin modification to form a strong binding through biotin-streptavidin interaction. Finally, quantum dot functionalized microspheres were washed with 0.1× PBS buffer 2–3 times to remove excess quantum dots from the solution. The QD-coated microspheres were used as prepared.

### Functionalization of the Gold Dimers with Cy5

The gold nanoparticle tipped post arrays were functionalized with Cy5 using thiol-modified 30 bp oligonucleotides (Integrated DNA Technologies). In order to form strong DNA brushes on the surface of gold nanoparticles, we first hybridized single stranded oligonucleotide strands into double-stranded DNA using a thermal cycler. The sequence of the oligonucleotide was carefully designed to minimize the chances for the self-dimerization (sequence 1: 5′–HS CCA TAG TCC TGC CCA ATC CTC TGT CGC CCA Cy5–3′; sequence 2: 5′–TGG GCG ACA GAG GAT TGG GCA GGA CTA TGG–3′). The equal molar amounts of two strands were annealed at 80 °C for 30 sec, and then gradually cooled to 25 °C (ramp rate 0.1 °C/sec) to hybridize. Throughout the functionalization, tris buffer (10 mM at pH 8) including 150 mM NaCl and 2 mM of MgCl_2_ was used.

After hybridization, the disulfide bonds of thiols in the DNA were reduced using immobilized TCEP disulfide reducing gel (Thermo Scientific Inc). For this, the TCEP gel (20 μL) was first washed with tris buffer for 3 times, mixed with DNA duplex (10 μL, 50 μM), and then incubated on a rocker for 1.5 hrs. After the TCEP gel was settled down, 10 μL of supernatant containing the reduced DNA duplex was carefully recovered from the mixture. The TCEP gel was further washed with tris buffer and supernatant recovery was repeated if DNA duplex needs concentration adjustments.

After the reduction of disulfide bonds of thiols in DNA, a drop of double-stranded 5′-thiolated Cy5-modified DNAs was added on the top surface of the prepared gold nanoparticle tipped post substrates and incubated in a dark, humid environment for overnight. After the incubation, the substrate was washed with tris buffer to remove unbound DNAs from the substrate. The gold nanoparticle dimer antennas showed an order of magnitude increase in fluorescence intensities compared to the same dyes spin-coated on the metal-free quartz substrates.

### Fluorescence Microscopy and Data Processing

Fluorescence images of the optoplasmonic structures were taken using an inverted optical microscope (Olympus IX71) equipped with an imaging CCD (Andor, iXon^EM^). A 420 nm LED (Thorlabs Inc.) was used as a light source to excite QD-605. The sample was mounted upside-down to directly excite the structure and to obtain the signal through the fluorescence filter sets (excitation/dichroic/emission) assembled for guiding (427/450/610 nm) and localization (427/662/680 nm) experiments. An additional long pass filter at 647 nm was added to the emission channel for the localization experiment to further reduce autofluorescence of the QD-coated OMs. The fluorescence images were resolved using a long-working distance 60× air objective (Olympus LUCPLFLN, NA = 0.70). For the guiding measurement the fluorescence images were taken using exposure times of 0.5 sec (10 time accumulation), and for the localization measurement the fluorescence images were taken at a kinetic mode using exposure times of 3 sec with a total of 25 kinetic series.

All fluorescence images were processed using custom-written Matlab codes (Ver. 7.11.0.584 R2010b; http://www.mathworks.com/products/matlab/). In order to select the precise location of the gold dimers and the OM junctions in the fluorescence image we compared the fluorescence images with SEM and optical microscopy images taken at the same area. In addition, we prepared a reference optoplasmonic chain that has the identical geometry as the sample. The selected areas of the gold dimers and the OM junctions (3 × 3 pixels each) in the reference image were precisely translated into the sample optoplasmonic chain to define and read the fluorescence intensity of the gold dimers and the OM junctions in the sample. This method ensured the accurate selection and measurement of the fluorescence intensities without being misguided by arbitrary area selection on the sometimes high background fluorescence images.

The maximum light power emitted by the QD coated microspheres is approximately 6 pW based on the incident light power (3.75 mW), the area of beam exposure (1.32 × 10^5^ μm^2^), the number of QDs bound to an individual OM (~1.40 × 10^3^), and the known extinction coefficient (2,800,000 M^−1^cm^−1^ at 405 nm) and quantum efficiencies (0.14 at 405 nm). The average number of QDs bound to individual PS beads was measured from high-resolution SEM images taken at magnifications >40 kX. Taking into account the emission pattern of the dyes and realistic in-coupling losses, we estimate that actual power fed into the optoplasmonic chain is <10 pW.

### FDTD Simulations

All FDTD simulations were performed using the Lumerical program (Ver. 8.7.0.; www.lumerical.com). The fitted data from Johnson and Christy was used for the refractive index of gold and a constant refractive index of n_r_ = 1.59 was applied for polystyrene beads. In the simulation of scattering cross sections, the total field scattered field (TFSF) source was used to illuminate the structure and a box of monitors surrounding the structure was included to record the scattering intensity. In the simulations of *E*-filed maps and Poynting vectors, emitting dipoles were used to simulate the quantum emitters. A mesh size of 30 nm was used in all three dimensions and fine meshes with mesh size of 5 nm were used at gold dimer and polystyrene bead junction positions. Electric filed intensities at each location were calculated by averaging the intensities of the whole area surrounded by the fine mesh and the pointing vectors were generated by computing the cross product of the electric and magnetic field. In all simulations, perfect matching layer (PML) was applied as the boundary condition. The *E*-field intensity distributions figures were generated using Matlab (Ver. 7.11.0.584 R2010b; http://www.mathworks.com/products/matlab/).

## Additional Information

**How to cite this article**: Ahn, W. *et al.* Low-Power Light Guiding and Localization in Optoplasmonic Chains Obtained by Directed Self-Assembly. *Sci. Rep.* **6**, 22621; doi: 10.1038/srep22621 (2016).

## Supplementary Material

Supplementary Information
