# Supplementary material for: Low-Power Light Guiding and Localization in Optoplasmonic Chains Obtained by Directed Self-Assembly
Source: Sci Rep. 2016 Mar 2;6:22621. doi: 10.1038/srep22621 (PMC4773872; doi:10.1038/srep22621)
Supplement: Supplementary Information [file srep22621-s1.pdf]

Low-Power Light Guiding and Localization in Optoplasmonic Chains Obtained by Directed Self-Assembly

*Wonmi Ahn, Xin Zhao, Yan Hong, and Björn M. Reinhard**

Department of Chemistry and The Photonics Center, Boston University, Boston, MA 02115, United States

**bmr@bu.edu*

Supporting Information

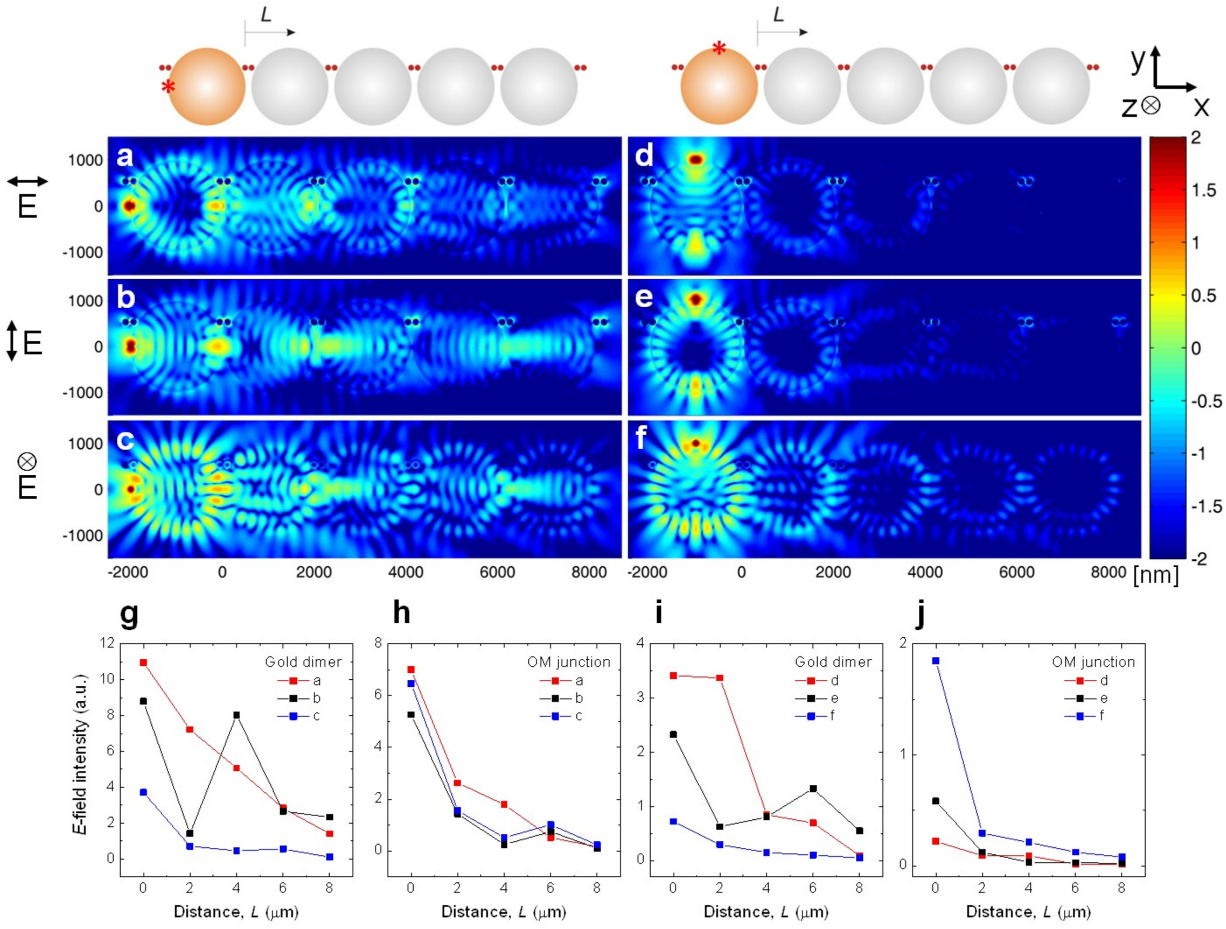

Figure S1. *E*-field maps of the optoplasmonic chains at diverse dipole emitter locations and orientations (a fixed wavelength of 650 nm). The dipole emitter is positioned on (a – c) and off (d – f) the chain axis; and is parallel to x- (a, d), y- (b, e), and z- (c, f) axes. The peak intensity values are obtained from 400 x 400 nm^2 areas centered at gold dimers and OM junctions and plotted in (g – j). *D* = 2 μm, *G* = 50 nm, *d* = 150, and *g* = 20 nm.

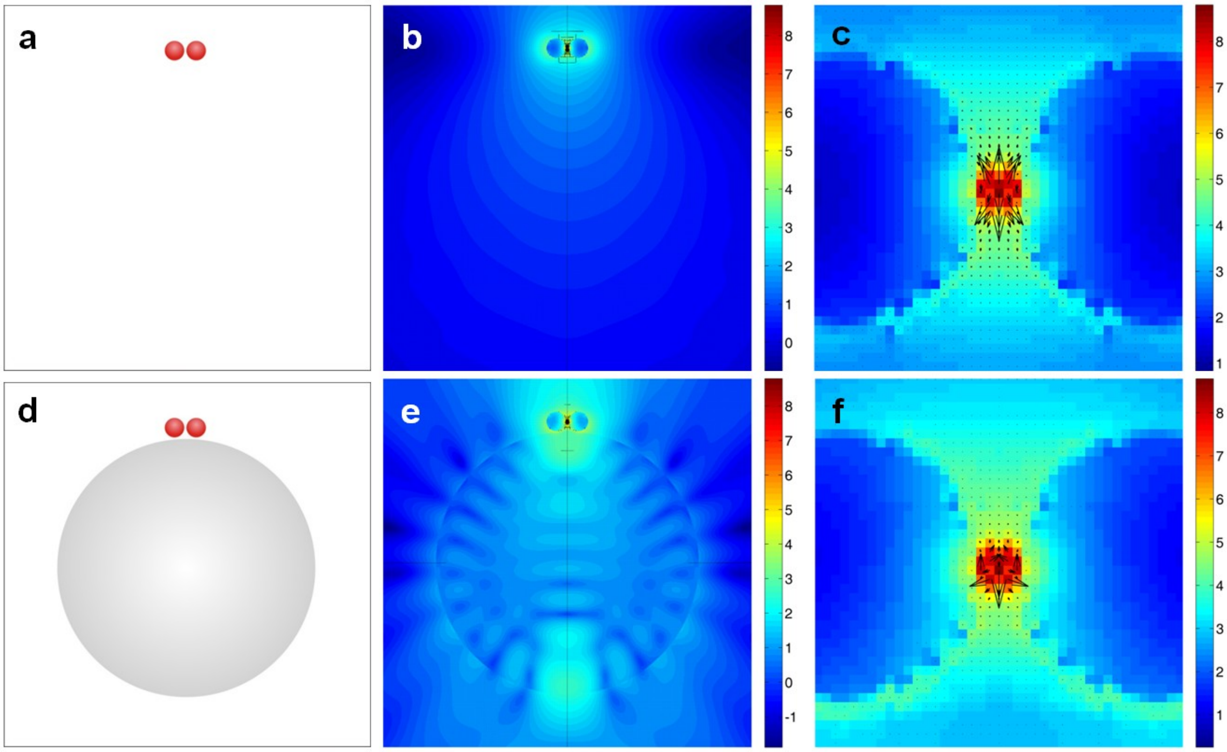

Figure S2. *E*-field and Poynting vector maps at λ = 680 nm for the gold dimer (*d* = 150 nm, *g* = 20 nm) in the absence (a – c) and presence (d – f) of the OM (*D* = 2 μm). (c) and (f) are gold dimers magnified from (b) and (e), respectively. Gold dimers are separated from the OM surface by 5 nm gaps.
